# Exploration of predictive risk factors for diabetic foot in patients with diabetes in Beijing: analysis of 5-year follow-up data of patients with diabetes mellitus in a single center in Beijing

**DOI:** 10.3389/fendo.2024.1441997

**Published:** 2024-08-07

**Authors:** Guanming Su, Xiaoyong Yuan, Geheng Yuan, Yalan Sun, Donghui Zhang, Wei Liu, Junqing Zhang, Xiaohui Guo

**Affiliations:** ^1^ Department of Endocrinology, Peking University First Hospital, Beijing, China; ^2^ Department of Endocrinology, The Second Hospital of Shijiazhuang, Shijiazhuang, China

**Keywords:** diabetes, diabetic foot, foot ulcer, risk factor, HbA1c

## Abstract

**Background:**

Large-scale prospective cohort studies on diabetic foot ulcers risk factor screening in China are limited. Therefore, this prospective cohort study aimed to explore the predictive risk factors for diabetic foot ulcers to provide clinicians with concise and effective clinical indicators for identifying a high-risk diabetic foot and guiding the prevention of diabetic foot ulcers.

**Methods:**

Patients with diabetes who visited the Department of Endocrinology of Peking University First Hospital from October 2017 to December 2018 were selected as research participants by convenience sampling. A total of 968 patients were included. After enrollment, a dedicated person collected and recorded all baseline data. A dedicated telephone follow-up was conducted every 12–24 months to evaluate whether the endpoint event had occurred. All patients were followed up for an average of 61 (57–71) months, with 95% of them followed up for more than 60 months. According to the occurrence of endpoint events, they were divided into the DFU and non-DFU groups. The data between the two groups were analyzed using independent-sample t-test, Wilcoxon rank sum test, and chi square test. We used univariate and multivariate logistic regression analysis to analyze the factors that affected the occurrence of diabetic foot ulcers.

**Results and conclusions:**

After the 5-year follow-up, the incidence of diabetic foot was 25.83%. Multivariate logistic regression analysis revealed that body mass index (odds ratio: 1.046; 95% confidence interval: 1.001–1.093), abnormal pinprick sensation (odds ratio: 4.138; 95% confidence interval: 1.292–13.255), history of fungal foot infection (odds ratio: 2.287; 95% confidence interval: 1.517–3.448), abnormal 128-Hz tuning fork test (odds ratio: 2.628; 95% confidence interval: 1.098–6.294), and HbA1c≥ 8% (odds ratio: 1.522; 95% confidence interval: 1.014–2.284) were independent predictors of diabetic foot. Our study highlights clinically relevant indicators that may help to prevent the occurrence of diabetic foot and guide timely interventions.

## 1 Introduction

Diabetic foot (DF) refers to the neuropathy and peripheral arterial lesions that can cause ulcers or damage to tissues far from the ankle, with or without infection. Diabetic foot ulcer (DFU) is the main manifestation of DF and one of the most serious complications of diabetes. The lifetime incidence rate of DFU can be as high as 19–34%, after successful healing the recurrence rate of diabetes-related foot ulceration is 40% within a year and 65% within 3 years (1). There are few studies on the incidence rate of DFU in China. Jiang et al. reported that the incidence of new ulcers in 1 year among patients with diabetes in China was 8.1%, and the incidence of recurrent ulcers in 1 year following healing of the initial DFU was 31.6% (2). DFU are harmful, with a poor prognosis and a high recurrence rate. They are one of the main reasons for amputation, disability, and death among patients with diabetes (3, 4). Hence, the 2023 International Working Group on the Diabetic Foot (IWGDF) guideline proposes that prevention is the most important step in the management of diabetic foot (5). Consequently, it is particularly important to find simple and effective DFU predictors to help clinicians identify high-risk diabetic foot (DFR) early and give timely individualized intervention programs. However, current studies on DFU risk factor screening, such as DFR screening systems and prediction models, are retrospective cohort studies, and there has been few prospective cohort study in China and large prospective cohort studies are lacked (6). This study aimed to explore predictive risk factors for DFU to provide clinicians with concise and effective clinical indicators for identifying DFR and promoting the prevention of DFU.

## 2 Materials and methods

### 2.1 Participants

**Figure 1:**
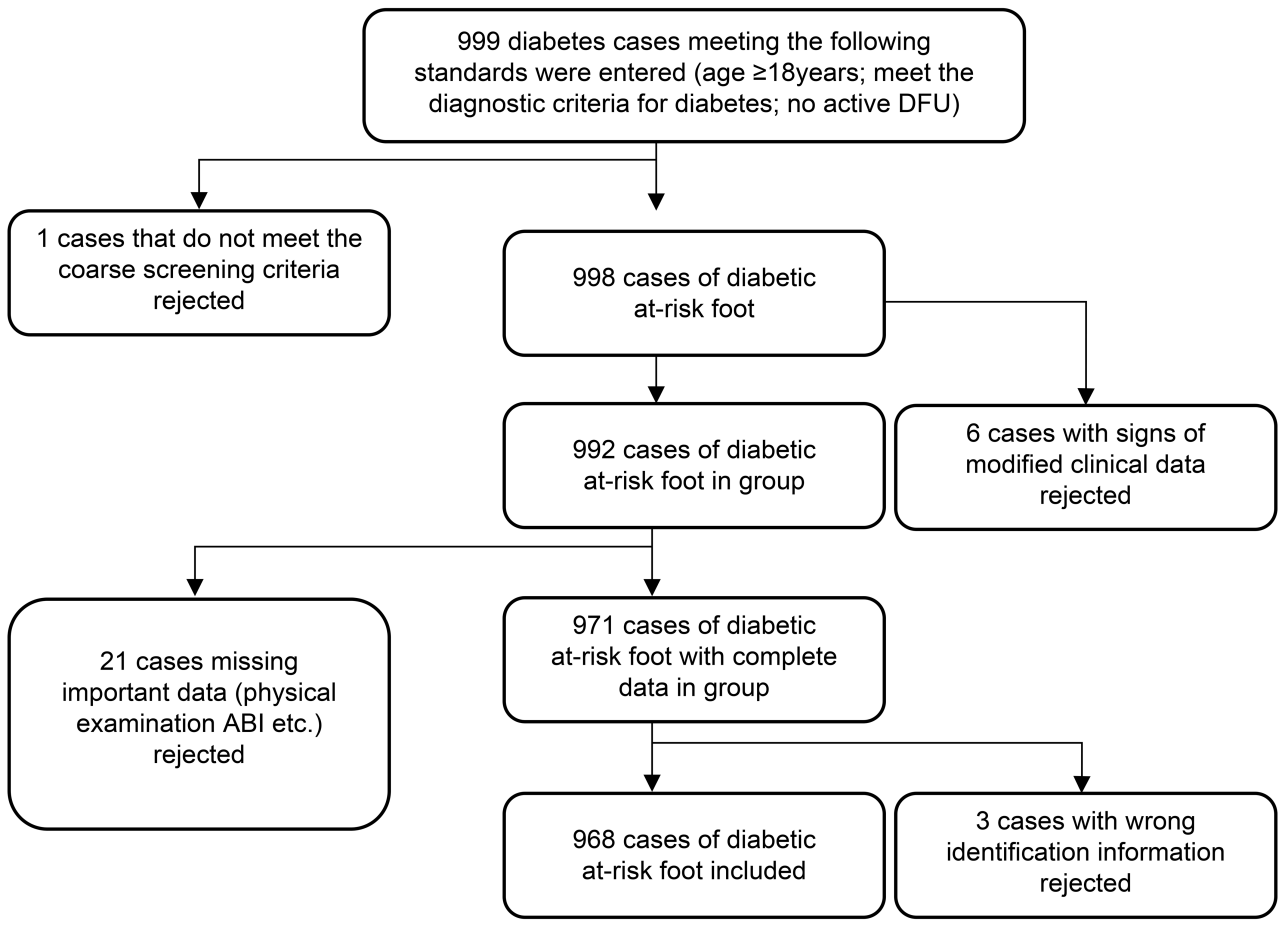
Flow chart of study participants.

Patients with diabetes who visited the Department of Endocrinology of Peking University First Hospital from October 2017 to December 2018 were selected as research participants through the convenience sampling method. The inclusion criteria were individuals aged ≥18 years who met the diagnostic criteria for diabetes and had no active DFU. The exclusion criteria were patients with existing foot ulcers, those who did not meet the coarse screening criteria, those with signs of modified clinical data or missing important data (physical examination results, ankle-brachial index [ABI], etc.), and those with wrong identification information. The coarse screening criteria included patients who met any of the following nine items: age more than 60 years; duration of diabetes more than 8 years; or a history of peripheral vascular disease, peripheral neuropathy (DPN), diabetic nephropathy, diabetic retinopathy, foot deformity, DFU and/or amputation, or smoking (pack-years≥20) (7). Accordingly, 968 participants were included in the study. The study was approved by the Medical Ethics Committee of Peking University First Hospital. Signed informed consent was obtained from all patients before participation (**Figure 1**).

### 2.2 Clinical data collection

Baseline data were collected from October 2017 to December 2018. The clinical data collection form was designed based on the factors associated with DFU in previous studies (8). The form included: general information such as sex, age, diabetes duration, smoking history, and history of fungal foot infection; past history and surgical history such as the presence of a comorbid condition (yes/no), hypertension, hyperlipidemia, hyperuricemia, chronic kidney disease, cardiovascular or cerebrovascular disease, peripheral vascular disease, surgical history of cardiovascular or cerebrovascular disease, history of lower extremity vascular surgery, and history of DFU/amputation; physical examination and laboratory indicators such as height, weight, body mass index (BMI), systolic blood pressure (SBP), diastolic blood pressure (DBP), fasting blood glucose (FBG), glycosylated hemoglobin (HbA1c), total cholesterol (CHOL), triglyceride (TG), high-density lipoprotein cholesterol (HDL-c), low-density lipoprotein cholesterol (LDL-c), urinary albumin/creatinine ratio (ACR), serum creatinine (Crea), and glomerular filtration rate (eGFR); and foot examination for abnormal changes in foot skin, foot fungal infections (tinea pedis, onychomycosis), foot deformities (hallux valgus, toe deformity, Charcot’s foot), calluses, intermittent claudication, rest pain, foot sensory disorders (numbness, prickling, formication, etc.), pulsation of the dorsal foot artery/posterior tibial artery, pressure sensation (10-g Semmes-Weinstein monofilament examination), temperature test, vibration sensation (128-Hz tuning fork test), pinprick test, ankle reflex, and ABI.

The medical history, physical examination, and foot examination were all inquired, examined, and recorded by trained diabetes specialists and nurses. The past history and surgical history were judged according to the patient’s previous outpatient or inpatient medical records in our hospital. If the pulsation of the dorsal foot artery/posterior tibial artery, 10-g Semmes-Weinstein monofilament examination, temperature test, 128-Hz tuning fork test, or pinprick test were abnormal on one side, it was regarded as abnormal. Abnormal bilateral ankle reflexes were considered abnormal. ABI was measured by an Omron BP-203RPEIII automatic blood pressure pulse wave detector. All laboratory test data were collected through the hospital’s outpatient digital medical record system and entered by a specially trained researcher. After input was completed, patient information was reviewed by a second researcher to ensure data accuracy. The following data were analyzed by grouping: age, less than 60 years or 60 years or more; diabetes duration, less than 10 years or 10 years or more; ACR, less than 30 mg/g (normal urine microalbumin group) or 30 mg/g or more (abnormal urine microalbumin group); HbA1c, less than 8.0% or 8.0% or more; and ABI, between 0.9-1.3 (normal) or 0.9 or less or more than 1.3 (abnormal).

### 2.3 Follow-up and diagnosis of DF

Follow-up was conducted from December 2019 to November 2023. The endpoint was the occurrence of DF. DF was defined as whether any foot had the following conditions after enrollment: new skin damage below the ankle with blood outflow; new blisters (scalds or abrasions) below the ankle; new skin infection below the ankle, including skin and soft tissue infection and/or bone infection; or new gangrene below the ankle. The diagnosis of DF was based on the Chinese guidelines for the prevention and treatment of Type 2 Diabetes (2017 Edition) (9). A dedicated telephone follow-up was conducted every 12–24 months to evaluate whether the endpoint event had occurred. If the endpoint had occurred, the predisposing factors for DF were inquired about, and the follow-up was stopped. Participants who could not be reached by telephone after three attempts were contacted in person, if possible, at the time of their scheduled clinic visit. According to the occurrence of endpoint events, patients were divided into the DFU and non-DFU groups.

### 2.4 Statistical analysis

All statistical analyses were performed using SPSS version 26.0 (Armonk, NY: IBM Corp). Measurements with normal distribution were analyzed using an independent-sample t-test, with results presented as means ± standard deviations. Nonnormally distributed data were analyzed using the Wilcoxon rank-sum test, with results presented as medians with interquartile ranges (Q1, Q3). Count data are expressed as numbers (percentages) and were analyzed using the χ² test. Univariate and multivariate logistic regression analyses were used to analyze factors influencing the occurrence of DFUs. Initially, univariate logistic regression was employed to screen potential influencing factors, which were then included in the multivariate logistic regression model to identify independent predictors. To ensure the robustness of the results, adjusted odds ratios (ORs) and their 95% confidence intervals (CIs) were calculated in the logistic regression models. All statistical tests were two-sided, with P values <0.05 considered statistically significant. Additionally, multicollinearity diagnostics were conducted to ensure that there were no highly collinear variables in the regression models.

## 3 Results

### 3.1 Participant characteristics

**Figure 2:**
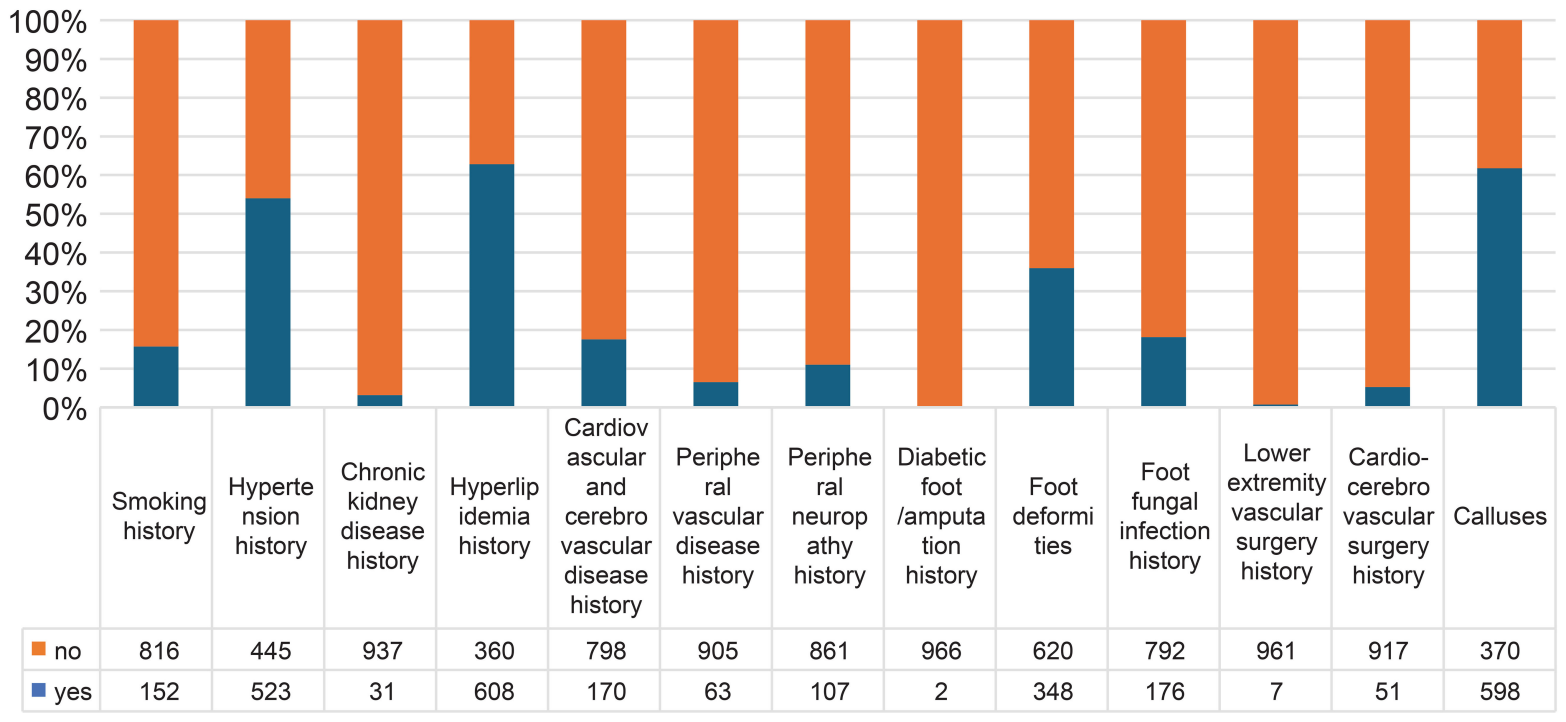
Baseline patient characteristics.

This study included a total of 968 patients with diabetes. The average age of the patients was 60.15 ± 11.37 years, and the average disease duration was 10.78 ± 8.03 years. In total, 544 patients (56.2%) were male, and 152 (15.7%) had a history of smoking. Regarding past history and past surgeries, 523 patients (54.0%) had hypertension, 31 (3.2%) had chronic kidney disease, 608 (62.8%) had hyperlipidemia, 170 (17.6%) had cardiovascular or cerebrovascular diseases, 63 (6.5%) had peripheral vascular diseases, 107 (11.1%) had DPN, 51 (5.3%) had a surgical history of cardiovascular or cerebrovascular disease, seven (0.7%) had a history of lower extremity vascular surgery, 176 (18.2%) had a history of foot fungal infections, two (0.2%) had a history of DFU/amputation, 348 (36.0%) had foot deformities, and 598 (61.8%) had calluses. All patients were followed up for an average of 61 months (57-71 months), with 95% of them followed up for more than 60 months. At the end of the 5-year follow-up, 250 patients (25.83%) had DFU, and 16 (1.7%) had died. There were 226 patients with DFUs of Wagner grade 1, 22 of grade 2, 0 of grade 3, 2 of grade 4, and 0 of grade 5 (**Figure 2**).

**Figure 3:**
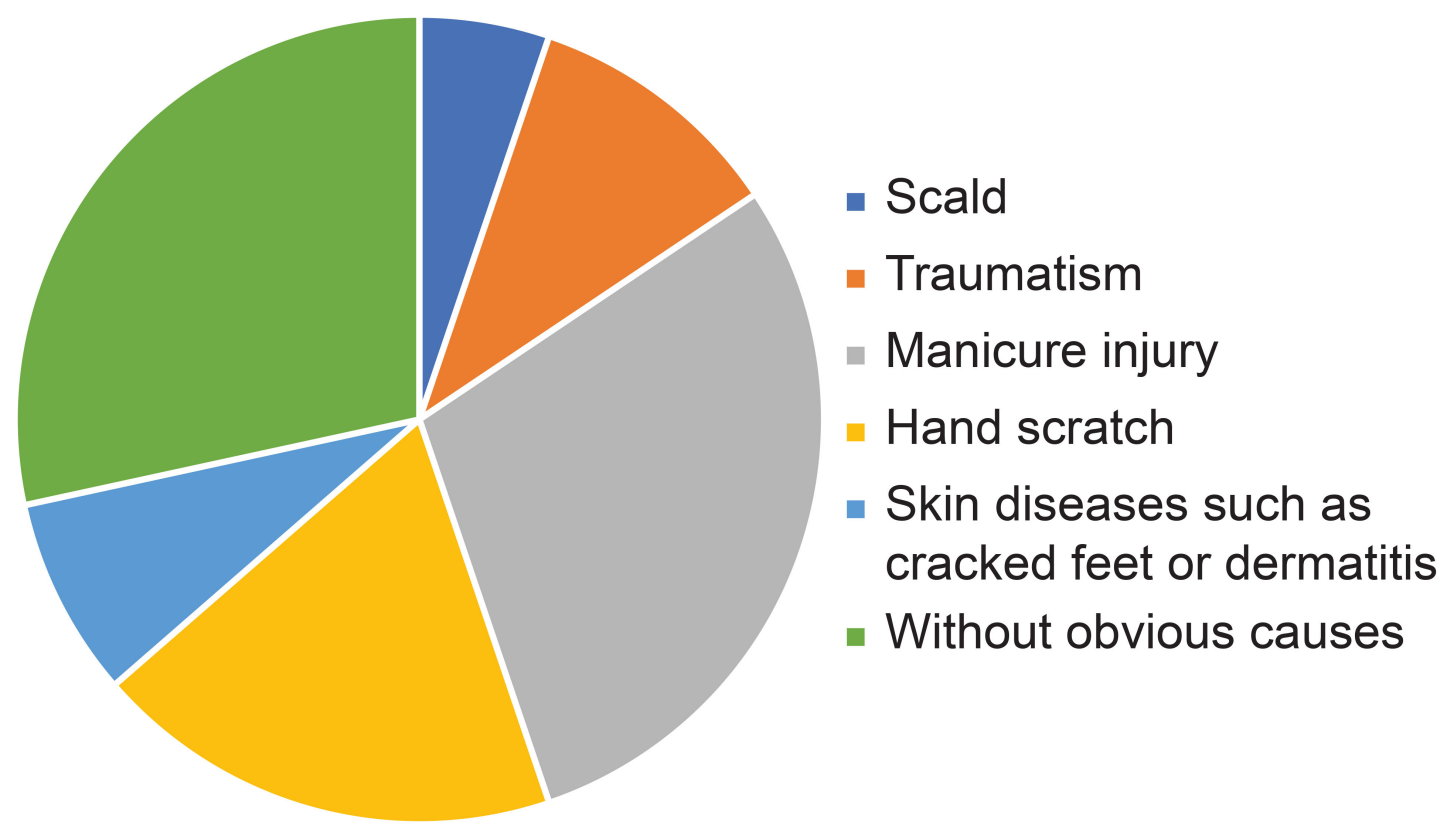
Proportion of diabetic foot ulcer causes.

The causes of DFU were scald (5.2%; 13/250), traumatism (10.4%; 26/250), manicure injury (29.2%; 73/250), hand scratch (18.8%; 47/250), skin diseases such as cracked feet or dermatitis (8%; 20/250), or without a definite cause (28.4%; 71/250) (**Figure 3**).

### 3.2 Comparison of baseline data between groups with and without DFUs

Measurement data between groups were compared with the t-test, and the differences in height, weight, and BMI were statistically significant (t = -2.587, -3.283, -2.310, respectively; *P* < 0.05). Enumerative data were compared with the χ2 test, and there were significant differences in abnormal pinprick sensation and history of foot fungal infection (*χ2 = *4.648, 25.545, respectively; *P* < 0.05). There were no statistically significant differences in the comparison of other baseline data between the two groups (all *P* > 0.05) (**Tables 1**, **2**).

**Table 1:** Comparison of the basic and inspection data between the groups with and without DFUs.

	DFU group	Non-DFU group	*t/z*	*P*
Number of patients Age (years)	250 59.53 ± 11.39	718 60.37 ± 11.37	1.011	0.313
Sex (male)	148 (59.2)	396 (55.2)	1.234	0.267
Diabetes duration (years)	11.05 ± 7.96	10.68 ± 8.07	-0.627	0.531
Height (cm)	167.78 ± 9.07	166.19 ± 8.17	-2.587	0.010
Weight (kg)	73.54 ± 13.87	70.39 ± 12.79	-3.283	0.001
BMI (*kg/m²*)	26.06 ± 4.35	25.40 ± 3.76	-2.310	0.021
SBP (mmHg)	133.39 ± 17.51	134.24 ± 17.54	0.661	0.509
DBP (mmHg)	80.07 ± 11.38	79.59 ± 10.60	-0.607	0.544
FBG (mmol/L)	7.72 ± 2.09	7.61 ± 2.08	-0.687	0.492
HbA1c (%)	7.39 ± 1.38	7.30 ± 1.34	-0.912	0.362
TG (mmol/L)	1.79 ± 1.98	1.65 ± 1.25	-1.343	0.180
CHOL (mmol/L)	4.23 ± 1.14	4.34 ± 1.08	1.279	0.201
HDL-c (mmol/L)	1.24 ± 0.85	1.23 ± 0.42	-0.244	0.807
LDL-c (mmol/L)	2.37 ± 0.77	2.44 ± 0.83	1.089	0.276
Crea (µmol/L)	86.12 ± 29.57	89.44 ± 68.43	0.713	0.476
ACR (mg/g)	8.69(4.435,25.16)	7.09(3.9275,17.2425)	-1.869	0.062
eGFR (mL/min/1.73 m²)	67.19 ± 17.34	67.87 ± 18.02	0.498	0.619

DFU, diabetic foot ulcer; BMI, body mass index; SBP, systolic blood pressure; DBP, diastolic blood pressure; FBG, fasting blood glucose; HbA1c, glycosylated hemoglobin; CHOL, total cholesterol; TG, triglyceride; HDL-c, high-density lipoprotein cholesterol; LDL-c, low-density lipoprotein cholesterol; ACR, urinary albumin/creatinine ratio; Crea, serum creatinine; eGFR, glomerular filtration rate.

**Table 2:** Comparison of past history and examination data between the groups with and without DFUs.

	DFU group n (%)	Non-DFU group n (%)	*χ^2^*	*P*
Number of patients Hypertension history	250 135 (54.0)	718 388 (54.1)	0.001	0.975
Hyperuricemia history	20 (8.0)	54 (7.5)	0.060	0.806
Hyperlipidemia history	163 (65.2)	445 (62.0)	0.824	0.364
Chronic kidney disease history	12 (4.8)	19 (2.6)	2.775	0.096
Peripheral vascular diseases history	18 (7.2)	45 (6.3)	0.265	0.607
Cardio-cerebrovascular diseases history	42 (16.8)	128 (17.8)	0.135	0.713
History of foot fungal infections	72 (28.8)	104 (14.5)	25.545	0.000
Smoking history	32 (12.8)	120 (16.7)	2.145	0.143
Cardio-cerebrovascular surgery history	10 (4.0)	41 (5.7)	1.087	0.297
Lower extremity vascular surgery history	3 (1.2)	4 (0.6)	0.360	0.549^#^
DFU/amputation history	1 (0.4)	1 (0.1)		0.450^*^
Foot sensory disorders	56 (22.4)	132 (18.4)	1.911	0.167
Intermittent claudication	7 (2.8)	21 (2.9)	0.010	0.919
Rest pain	7 (2.8)	17 (2.4)	0.143	0.705
Foot deformities	84 (33.6)	264 (36.8)	0.809	0.369
Calluses	149 (59.6)	449 (62.5)	0.676	0.411
10-g Semmes-Weinstein monofilament examination	15 (6.0)	31 (4.3)	1.150	0.284
Temperature test	14 (5.6)	43 (6.0)	0.051	0.822
Abnormal 128-Hz tuning fork test	13 (5.2)	23 (3.2)	2.065	0.151
Abnormal pinprick sensation	8 (3.2)	7 (1.0)	4.648	0.031^#^
Ankle reflex	29 (11.6)	84 (11.7)	0.002	0.966
Pulsation of the dorsal foot/posterior tibial artery	38 (15.2)	111 (15.5)	0.010	0.922
ABI Grouping (Abnormal)	39 (15.6)	120 (16.7)	0.148	0.700

*Fisher’s exact tests; #Continuity-corrected chi-square.

DFU, diabetic foot ulcer; ABI, ankle brachial index.

### 3.3 Univariate logistic regression analysis for the risk of DFUs

The univariate logistic regression analysis was performed with the occurrence of DFU during the follow-up period as the dependent variable and various clinical indicators as independent variables. Age, diabetes duration, ACR, HbA1c level, and ABI were grouped and included as independent variables. Five factors associated with DFU were identified. These included a history of foot fungal infection, abnormal pinprick sensation, BMI, height, and weight, which were all positively correlated with DFU (*Wald* = 24.641, 5.366, 5.146, 6.624, 10.487, respectively; *P* < 0.05) (**Table 3**).

**Table 3:** Results of the univariate logistic regression analysis.

	*β*	SE	*Wald*	*P*	OR	95% CI
Sex	-0.165	0.149	1.232	0.267	0.848	0.633–1.135
Age Grouping (≥60years)	-0.050	0.148	0.113	0.737	0.951	0.712–1.271
Duration Grouping (≥10 years)	0.089	0.098	0.819	0.365	1.093	0.902–1.325
Hypertension history	-0.005	0.147	0.001	0.975	0.995	0.746–1.329
Hyperlipidemia history	0.139	0.153	0.824	0.364	1.149	0.851–1.553
Cardio-cerebrovascular diseases history	-0.072	0.195	0.135	0.713	0.931	0.635–1.365
DFU/amputation history	1.058	1.416	0.558	0.455	2.880	0.179–46.209
History of foot fungal infection	0.870	0.175	24.641	0.000	2.388	1.693–3.368
Smoking history	-0.313	0.214	2.132	0.144	0.731	0.481–1.113
Height	0.023	0.009	6.624	0.010	1.023	1.006–1.041
Weight	0.018	0.006	10.487	0.001	1.018	1.007–1.029
BMI	0.041	0.018	5.146	0.023	1.042	1.006–1.080
TG	0.060	0.047	1.670	0.196	1.062	0.969–1.164
LDL-c	-0.101	0.093	1.186	0.276	0.904	0.754–1.084
EGFR	-0.002	0.004	0.248	0.618	0.998	0.989–1.006
ACR Grouping (≥ 30 mg/g)	0.271	0.198	1.874	0.171	1.312	0.889–1.934
HbA1c (≥8%)	0.216	0.170	1.611	0.204	1.241	0.889–1.732
Foot deformities	-0.139	0.155	0.808	0.369	0.870	0.643–1.178
Foot sensory disorders	0.248	0.180	1.905	0.168	1.281	0.901–1.823
Calluses	-0.123	0.150	0.676	0.411	0.884	0.658–1.186
Intermittent claudication/rest pain	0.010	0.331	0.001	0.977	1.010	0.528–1.932
Pulsation of the dorsal foot/posterior tibial artery	-0.020	0.204	0.010	0.922	0.980	0.657–1.463
ABI Grouping (Abnormal)	-0.077	0.201	0.148	0.700	0.925	0.624–1.372
Abnormal pinprick sensation	1.211	0.523	5.366	0.021	3.358	1.205–9.357
Temperature test	-0.071	0.317	0.051	0.822	0.931	0.500–1.733
Abnormal 128-Hz tuning fork test	0.505	0.355	2.026	0.155	1.657	0.826–3.324
10-g Semmes-Weinstein monofilament examination	0.345	0.323	1.140	0.286	1.412	0.749-2.663
Ankle reflex	-0.010	0.229	0.002	0.966	0.990	0.632-1.552

DFU, diabetic foot ulcer; OR, odds ratio; CI, confidence interval; BMI, body mass index; HbA1c, glycosylated hemoglobin; TG, triglyceride; LDL-c, low-density lipoprotein cholesterol; ACR, urinary albumin/creatinine ratio; eGFR, glomerular filtration rate; ABI, ankle-brachial index; SE, standard error.

### 3.4 Multivariate logistic regression analysis for the risk of DFUs

**Figure 4:**
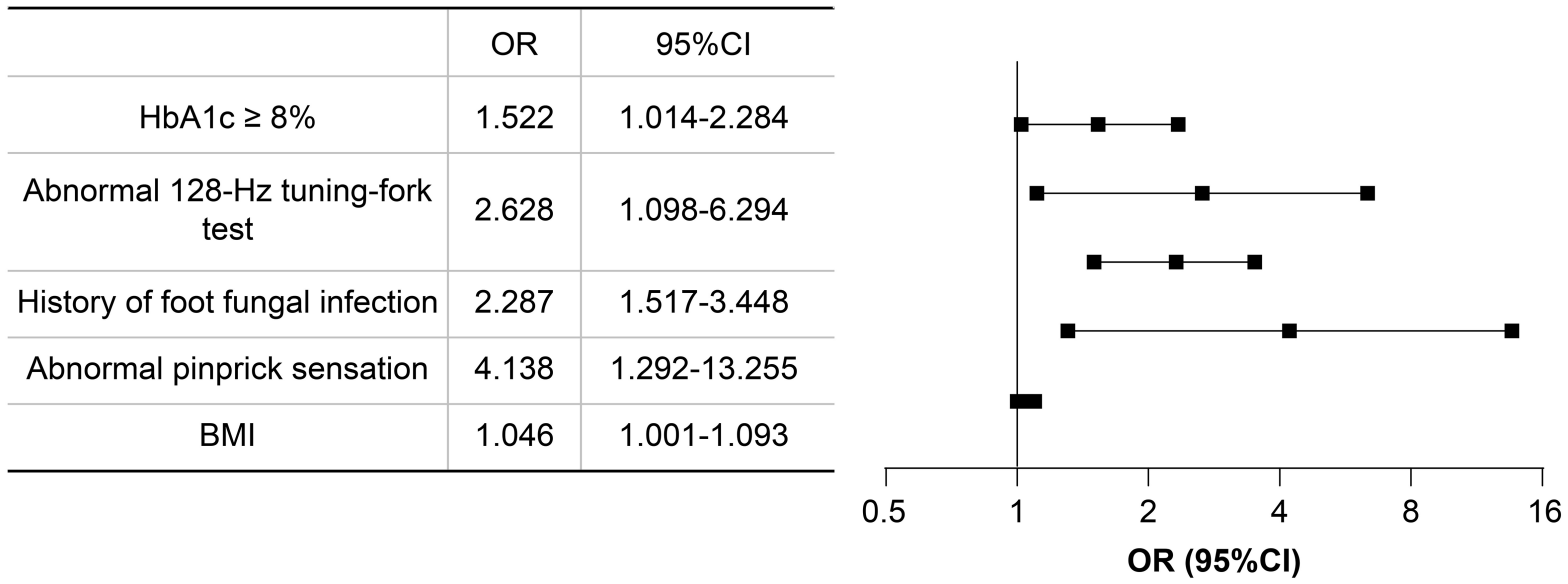
Forest plot of multivariate logistics regression analysis. CI, confidence interval; OR, odds ratio.

**Figure 5:**
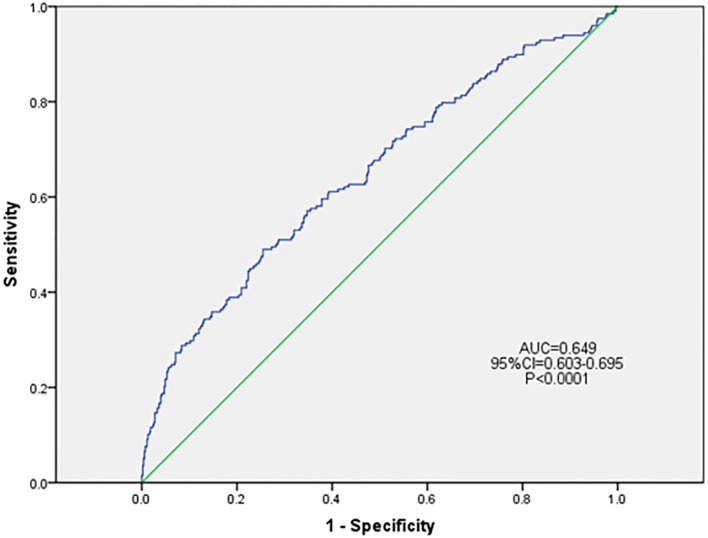
ROC curve of the risk prediction model for DFU. DFU, diabetic foot ulcer; CI, confidence interval; ROC, receiver operating characteristic; AUC, area under the ROC.

Based on the results of univariate logistic regression analysis, clinical logic relationships in our study and previous literature, the following 26 factors were selected as independent variables for multivariate logistic regression analysis: sex, age grouping(≥60 years), duration grouping (≥10 years), HbA1c≥8%, BMI, TG, LDL-c, EGFR, ACR grouping (≥30 mg/g), smoking history, foot deformities, foot sensory disorders, calluses, intermittent claudication/rest pain, pulsation of the dorsal foot/posterior tibial artery, ABI (abnormal), abnormal pinprick sensation, temperature test, abnormal 128-Hz tuning fork test, 10-g Semmes–Weinstein monofilament examination, ankle reflex, history of DFU/amputation, hypertension history, hyperlipidemia history, cardio-cerebrovascular diseases history, and history of foot fungal infection. Height and weight were excluded because BMI was calculated based on them. The results showed that BMI (odds ratio [OR]: 1.046; 95% confidence interval [CI] 1.001–1.093), abnormal pinprick sensation (OR: 4.138; 95% CI: 1.292–13.255), history of foot fungal infection (OR: 2.287; 95% CI: 1.517–3.448), abnormal 128-Hz tuning fork test (OR: 2.628; 95% CI: 1.098–6.294), and HbA1c≥ 8% (OR: 1.522; 95% CI: 1.014–2.284) were independent predictors of DFUs. Hosmer–Lemeshow goodness-of-fit test showed that *χ2 = *7.640; *P* > 0.05. The collinearity diagnosis results show that the VIF of all independent variables is less than 10. Receiver operating characteristic (ROC) curve analysis was performed with DF as the dependent variable and predicted probability as the independent variable. The results showed that the area under the ROC curve (AUC) was 0.649 (95% CI: 0.603–0.695; P < 0.0001), the sensitivity was 0.49, the specificity was 0.745. (**Table 4**, **Figures 4**, **5**).

**Table 4:** Results of the multivariate logistic regression analysis.

	*β*	*SE*	*Wald*	*P*	*OR*	*95% CI*
BMI	0.045	0.022	4.093	0.043	1.046	1.001–1.093
Abnormal pinprick sensation	1.420	0.594	5.719	0.017	4.138	1.292–13.255
History of foot fungal infection	0.827	0.210	15.582	0.000	2.287	1.517–3.448
Abnormal 128-Hz tuning fork test	0.966	0.446	4.705	0.030	2.628	1.098–6.294
HbA1c ≥ 8%	0.420	0.207	4.099	0.043	1.522	1.014–2.284

OR, odds ratio; CI, confidence interval; BMI, body mass index; HbA1c, glycosylated hemoglobin; SE, standard error.

## 4 Discussion

DFU is one of the most serious complications of diabetes mellitus. Its lifetime risk is 19–34%, with an annual growth rate of 2% (1). The incidence of DFU is not consistent across studies. Studies from 2006 to 2019 found that the lifetime risk of DFU in patients with diabetes is approximately 15–25% (10, 11). The present study showed that the 5-year incidence of DFU in patients from Beijing is 25.83%, higher than that reported in previous studies. This may have been related to the baseline number of patients and follow-up time. The patients enrolled in our study were screened using the crude screening criteria for DFR. These participants were classified as high risk foot 0+. It indicates that the coarse screening index of diabetic foot mentioned by Professor Xiaohui Guo (7) in “The Standardized Process of Screening, classification and Intervention of Diabetic patients with high-risk foot” can achieve the purpose of primary screening of DFR. Moreover, the 5-year follow-up period in our study may have contributed to the higher incidence of DFU compared with that in other studies.

DFU, characterized by foot ulcers with or without microbial infection, is a major cause of lower limb amputation. DPN and peripheral artery disease (PAD) are risk factors for DFUs. DFU combined with DPN and PAD is called DFR; however, the clinical symptoms of DFR are not typical, making it difficult to detect by routine examination. Implementation of the DFR rapid screening model reduces the risk of DFUs (12) and can reduce the number of amputations. It is particularly important to simply, effectively, and economically screen for patients with DFR and intervene early to prevent DFUs.

In the IWGDF 2019 guidelines, individuals with a loss of protective sensation (LOPS) have an increased risk for ulcers (1, 8). LOPS can be detected with tests such as the 128-Hz tuning fork test, 10-g Semmes–Weinstein monofilament examination, and vibration perception threshold (VPT) meter. In this study, an abnormal 128-Hz tuning fork test was found to be an independent risk factor for DFUs. Xie et al. (13) showed that patients with a VPT of more than 25v had a significantly increased risk of DFUs, emphasizing the significance of VPT for the prevention of DFUs. A comparison of the detection performance of the 128-Hz tuning fork test and 10-g Semmes–Weinstein monofilament examination with the VPT showed that the specificity of both detections was 78.9%, with a positive correlation with VPT (14). However, compared with the VPT, the 128Hz tuning fork test is more concise, effective, and cost-effective in clinical work. Our study failed to find an association between an abnormal 10 g monofilament examination and DFUs. The 10 g monofilament examination is economical, quick, and convenient to use; several studies have demonstrated its feasibility as a risk factor for predicting DFUs. However, there is no consensus on the detection location and number of 10-g Semmes–Weinstein monofilament examinations (15). The 10-g filament test has varied locations and number of tests performed in several studies (16, 17); moreover, it is easy to be affected by callus, resulting in decreased sensitivity. If the 10-g Semmes–Weinstein monofilament examination is promoted in the early screening of DFR in primary medical and health institutions, standardized training is needed.

The pinprick sensation examination can evaluate small fiber neuropathy, and an abnormal pinprick sensation was found to be an independent risk factor for DFUs. Nerve damage initially occurs in unmyelinated and thin-myelinated sensory small-fiber nerves (Aδ-fibers) and autonomic small-fiber nerves (C-fibers) (18). Research has found that small fiber neuropathy may be related to the development of foot ulcers (19). A prospective study (20) used a pinprick test to determine the presence of loss of protective pain perception (LOPP) and found that a significant positive correlation existed between abnormal pinprick sensation and DFUs. When LOPP and LOPS coexist, the DFU occurrence time is significantly shorter in patients with LOPS without LOPP. To the best of our knowledge, this is the first prospective study in a Chinese population to find an association between LOPP and DFU. We believe that LOPP through acupuncture may increase the sensitivity of early identification of DFR.

Metabolic abnormality is the basic change seen in diabetes, such as hyperglycemia, insulin resistance, hypertension, dyslipidemia, and obesity. This change makes patients with diabetes more prone to atherosclerosis and consequently, PAD, increasing their risk of ulcers and infections. It is reported that 30–78% of patients with DFU have PAD (5, 21, 22). In the present study, we included the common clinical metabolic indicators leading to atherosclerosis, including BMI, HbA1c, TG, and LDL-c. Our results showed that BMI and an HbA1c level of 8% or more were independent risk factors for DFUs. Multiple studies (23, 24) have reported that HbA1c is an independent risk factor for predicting the occurrence of DFUs; however, the HbA1c cut values vary among studies. One study showed that patients with an average HbA1c level of more than 9% were more likely to have DFUs (25). Another study reported a 371% increased risk of DFU in patients with a HbA1c level of ≥8% compared with patients with a HbA1c level of <7% (26). Farooque et al. demonstrated a linear relationship between HbA1c level and Wagner grades, with higher HbA1c values indicating a higher Wagner grade. The HbA1c level associated with patients with Wagner grades 4 and 5 is more than 8.5%, while a vast majority of patients with Wagner grades 2 and 3 have a HbA1c level ranging from 7.5% to 8.5% (27). In this study, HbA1c levels of 7.0%, 7.5%, 8.0%, and 8.5% were used as cut points for analysis, and the ORs and P-values of the multivariate logistic regression analysis were: 1.189 and 0.330 for a HbA1c of 7.0%, 1.167 and 0.418 for a HbA1c of 7.5%, 1.522 and 0.043 for a HbA1c of 8.0%, and 1.473 and 0.097 for a HbA1c of 8.5%. This suggests that a HbA1c of 8% may be a more sensitive and specific blood glucose cut-off point for DFU risk.

BMI is a simple and effective index reflecting a patient’s weight status. In this study, a positive correlation was observed between high BMI and DFUs. In 2023, Lv et al. first included a high BMI as one of the indicators for predicting DFUs in their research (28). Another meta-analysis showed that a BMI of more than 24.5 is an independent risk factor for newly diagnosed DFUs (29). Traditionally, patients with DFUs have a higher risk of amputation and death with a lower BMI than those with a higher BMI. Undernourished patients with DFUs are considered to have more foot ulcers, higher Wagner grades, and higher amputation rates (30). However, in patients who do not yet have a DFU, a high BMI is often accompanied by metabolic abnormalities that increase the risk of atherosclerosis, which in turn leads to vascular stenosis in the lower limbs, resulting in lower-limb ischemia and foot ulcers (31). Moreover, a high BMI increases plantar pressure, and studies have shown that the higher the plantar pressure, the higher the risk of foot ulcers (32, 33). Patients with increased foot pressure have a two-fold increased risk of foot ulcers (34).

No association was found between TG and LDL-c levels and DFUs. Previous studies (22, 35) have shown that LDL-c and HDL-c levels may be high-risk factors for the occurrence of DFUs; however, we failed to find a significant correlation. This may have been due to the fact that 62.8% of the participants had hyperlipidemia; hence, the long-term use of lipid-lowering drugs may have affected the outcomes.

This study has some limitations. First, it was difficult to control the quality of data by collecting endpoint events through telephone follow-up. Second, the sample size of this study was small, and it was a single-center cohort study. Third, there was a selection bias among the enrolled patients as they needed to meet the coarse screening criteria). Therefore, future studies are needed with larger sample sizes, multi-center participation, and the inclusion of all diabetes patients without active ulcers to further validate our findings. Follow-up studies are needed to continue refining the diagnostic model.

In conclusion, the incidence of DFUs in patients with diabetes was found to be 25.83% within 5 years. Moreover, BMI, abnormal pinprick sensation, history of foot fungal infection, abnormal 128-Hz tuning-fork test, and a HbA1c level of 8% or more were independent predictors of DFUs. These results may provide clinicians with concise and effective indicators for identifying DFR, thereby enabling them to provide appropriate help for the early prevention of DFUs. As the specificity is much greater than the sensitivity, it indicates that it is more likely to rule out the possibilities of DFU for patients who tested negative. Conversely, patients who tested positive may need further diagnostic tests to confirm whether they were affected by DFU.

## Data availability statement

The raw data supporting the conclusions of this article will be made available by the authors, without undue reservation.

## Ethics statement

The studies involving humans were approved by Medical Ethics Committee of Peking University First Hospital Peking University First Hospital. The studies were conducted in accordance with the local legislation and institutional requirements. The participants provided their written informed consent to participate in this study.

## Author contributions

GS: Writing – original draft, Writing – review & editing, Data curation, Formal Analysis, Conceptualization, Investigation, Visualization. XY: Writing – original draft, Writing – review & editing, Conceptualization, Methodology, Project administration, Supervision, Validation, Software. GY: Supervision, Writing – review & editing, Project administration, Resources. YS: Investigation, Writing – review & editing. DZ: Investigation, Writing – review & editing. WL: Investigation, Writing – review & editing. JZ: Supervision, Writing – review & editing, Funding acquisition. XG: Supervision, Writing – review & editing, Funding acquisition, Project administration, Conceptualization.
